# Beyond Classic Leprosy: Exploring Atypical Manifestations and Their Diagnostic Challenges

**DOI:** 10.7759/cureus.75957

**Published:** 2024-12-18

**Authors:** Aruna Bathina, Haritha Kollipara, Gedela Sravani, Vijaya Sree Laxmi

**Affiliations:** 1 Department of Dermatology, Venereology and Leprosy, Great Eastern Medical School & Hospital, Srikakulam, IND; 2 Department of Dermatology, Venereology and Leprosy, Gandhi Institute of Technology and Management Institute of Medical Sciences and Research, Visakhapatnam, IND

**Keywords:** erythema nodosum leprosum (enl), granuloma annulare, hansen’s disease, histoid leprosy, leprosy, lucio phenomenon, multidrug therapy (mdt), pure neuritic leprosy, sweet’s syndrome

## Abstract

Leprosy, or Hansen’s disease, is an ancient infectious disease characterized by varied clinical presentations influenced by the host's immune response. This study aimed to explore the atypical manifestations of Hansen’s disease in a cohort of 15 biopsy-confirmed patients admitted to the Department of Dermatology, Venereology & Leprosy at a tertiary care center in Andhra Pradesh, India. There were 14 male patients and one female patient, with a mean age of 42.8 years. Notably, six patients presented with erythema nodosum leprosum (ENL), showing diverse atypical manifestations such as Sweet’s syndrome-like lesions, Lucio phenomenon, and erythema nodosum necroticans. Two patients were classified under the histoid spectrum with multiple asymptomatic nodular lesions, while three exhibited tuberculoid features mimicking conditions like granuloma annulare and urticarial vasculitis. Additionally, two cases of pure neuritic Hansen’s disease were observed, presenting with isolated nerve involvement and significant sensory impairment. The last two cases presented with lepromatous spectrum-simulating erythromelalgia and spontaneous ulceration of both hands.

This study aims to address the diagnostic challenges posed by atypical presentations of leprosy, which often lead to delays in diagnosis and, in some cases, misdiagnosis. Initially, all the patients exhibited symptoms that were not typical of classic leprosy, resulting in diagnostic delays. After histopathological confirmation, they were treated with multidrug therapy (MDT), with systemic steroids added for those with ENL to manage severe inflammatory responses. This study emphasizes the critical importance of clinical awareness and early detection, particularly in endemic regions, to facilitate prompt intervention. Timely recognition and treatment are essential to interrupt the transmission cycle and progress toward a leprosy-free world.

## Introduction

Leprosy, also known as Hansen's disease, is one of the world's oldest recognized diseases, with a documented presence in ancient civilizations such as Egypt, China, and India. Historically, leprosy has been surrounded by fear and misunderstanding, resulting in severe social stigma and isolation of the affected individuals [1-4]. The disease is caused by two bacteria: Mycobacterium leprae and Mycobacterium lepromatosis [5-7].

In India, seven states, i.e., Bihar, Uttar Pradesh, Chhattisgarh, Jharkhand, Maharashtra, Odisha, and West Bengal, account for nearly three-quarters of the country's total leprosy burden [8,9]. Although India has officially achieved the goal of leprosy elimination, the disease remains a major public health concern in hyperendemic regions. As of 2021-22, the prevalence rate stands at 0.45 cases per 10,000 population [10]. The annual new case detection rate has also declined, dropping from 9.73 per 100,000 population in 2014-15 to 5.52 in 2021-22 [10].

Despite significant progress in reducing leprosy's impact, the emergence of atypical clinical presentations poses an ongoing challenge. Leprosy primarily affects the skin and peripheral nerves, but in cases with high bacillary load, internal organs may also be involved. Uncommon manifestations, such as erythema nodosum leprosum (ENL), can present as vesiculobullous lesions, Sweet's syndrome-like conditions, erythema multiforme-like reactions, Lucio phenomenon (LP), and reactive perforating types [11]. These atypical variants often mimic other dermatological diseases, complicating accurate diagnosis.

Our study focuses on the diagnostic challenges associated with atypical presentations of leprosy, which frequently result in delayed diagnoses and, in some cases, incorrect diagnoses. The patients in this study initially presented with symptoms that deviated from those typically observed in classic leprosy, complicating the diagnostic process. After confirmation through histopathological analysis, all patients were started on multidrug therapy (MDT) as the standard treatment. Additionally, for patients experiencing ENL, systemic steroids were prescribed to manage severe inflammatory responses effectively.

The findings underscore the need for heightened clinical awareness and vigilance, especially in regions where leprosy remains endemic. Atypical manifestations of Hansen’s disease can obscure its identification, leading to delays in initiating appropriate treatment. These delays not only exacerbate the disease’s progression in the affected individuals but also increase the risk of transmission within the community.

This study highlights the critical importance of equipping healthcare professionals with the knowledge and tools to recognize less common presentations of leprosy promptly. Enhanced training and awareness can lead to earlier detection, timely intervention, and better patient outcomes. Furthermore, breaking the transmission chain through early diagnosis and treatment is vital in the global effort to eliminate leprosy and work toward a leprosy-free future.

## Materials and methods

Study design

Type of Study

This retrospective study, conducted over one year from April 2023 to April 2024, focused on biopsy-confirmed cases of Hansen’s disease with atypical presentations. It specifically targeted patients who exhibited atypical dermatological manifestations and lacked the classic cardinal signs of leprosy. These unusual features often mimicked other dermatological or systemic conditions, making comprehensive diagnostic evaluations essential to establish an accurate diagnosis.

Location

The study was conducted in the Dermatology, Venereology, and Leprosy (DVL) outpatient department of a tertiary care center in Andhra Pradesh, India. Data were gathered from the medical records of Hansen’s disease cases managed in the DVL department, as well as from referrals received from other specialties, including general surgery, neurology, pulmonology, and others. This multidisciplinary approach ensured a comprehensive collection of cases with diverse clinical presentations.

Clinical and Laboratory Data

The variables collected for the study included demographic information such as age, sex, occupation, and disease duration. Clinical data encompassed presenting symptoms, detailed patient history, clinical features including systemic manifestations, and findings from comprehensive cutaneous examinations. Additionally, investigations performed included hematological, biochemical, and serological tests; slit skin smear analysis; biopsy results; and the treatment protocols administered.

Inclusion and Exclusion Criteria

The inclusion criteria for the study were designed to ensure a focus on atypical presentations of Hansen’s disease. Firstly, only cases of leprosy that were confirmed through histopathological evaluation (biopsy-confirmed diagnosis) were included. Secondly, it included patients who exhibited atypical dermatological manifestations, such as erythematous nodules, urticarial plaques, vesiculobullous lesions, or other unusual skin presentations that mimicked dermatological conditions like Sweet’s syndrome, erythema multiforme, or Lucio phenomenon.

Patients with classic clinical and histopathological features of polar tuberculoid, dimorphous, or lepromatous leprosy [e.g. Ridley-Jopling tuberculoid (TT), borderline tuberculoid (BT), mid borderline (BB), borderline lepromatous (BL), and lepromatous leprosy (LL) types] were excluded from the study. Cases lacking biopsy confirmation of Hansen’s disease were also excluded. 

Ethical approval for the study was obtained from the institutional ethics committee of the Great Eastern Medical School & Hospital, Srikakulam, India (Approval No.: 93/IEC/GEMS&H/ dated 13/04/2024). Written informed consent was collected from all patients before their inclusion in the study.

The analysis focused on describing the frequency and types of atypical presentations of Hansen’s disease, as well as associated systemic involvement. This approach highlighted the diagnostic challenges encountered and offered valuable insights into early detection and tailored treatment strategies for atypical cases.

## Results

**Figure 1:**
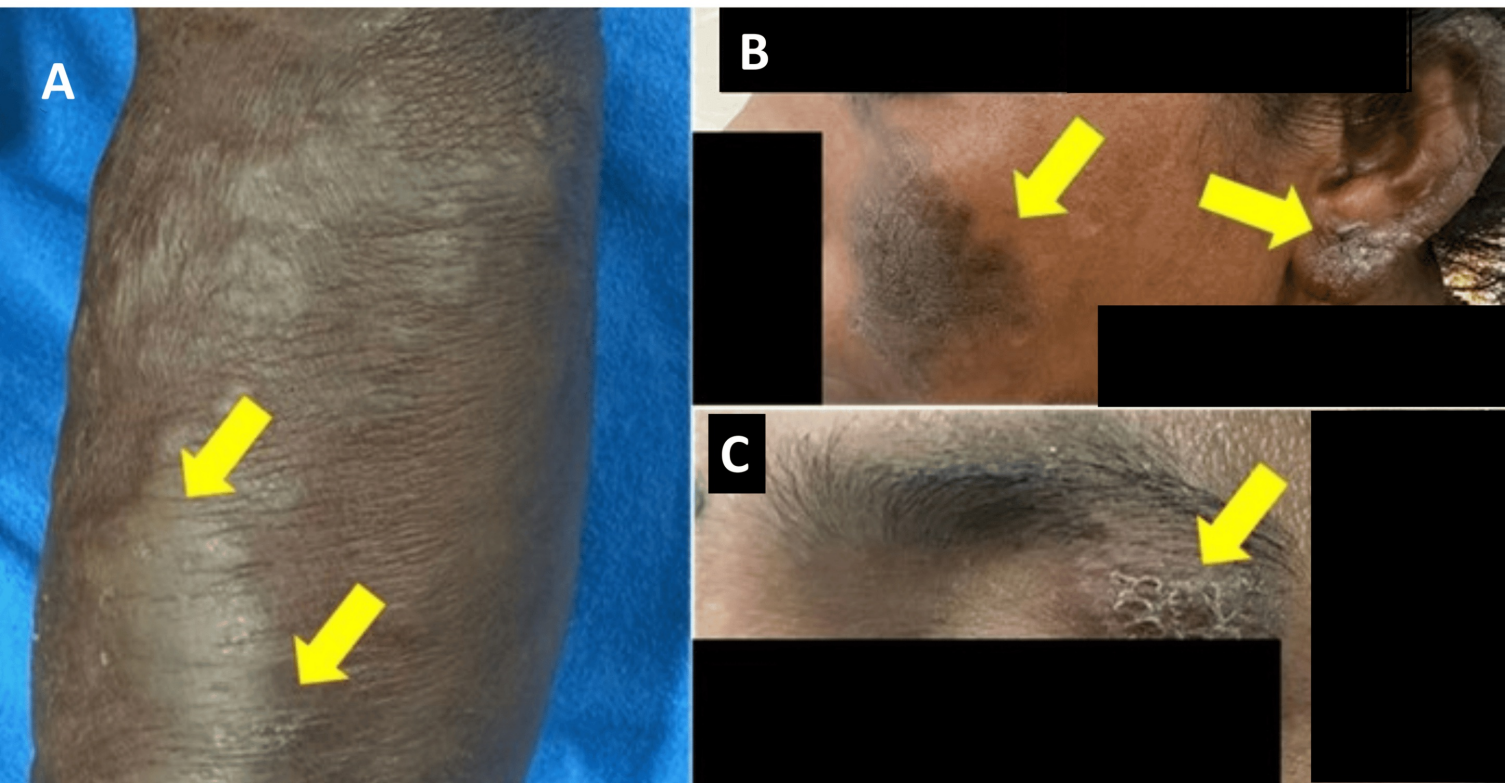
Hansen’s disease A) Hyperpigmented scaly indurated plaques over the dorsal aspect of the left hand, B) earlobe, C) eyebrow and malar area.

In this study of 15 biopsy-confirmed cases of Hansen’s disease with atypical symptoms, 14 male patients and one female patient were included, with a mean age of 42.8 years. One case had hyperpigmented (anesthetic) scaly indurated plaques on the hands, malar area, eyebrows, and earlobes (Figure 1).

Some of these patients had atypical skin presentations in addition to fever and joint pains. The characteristics of all the cases are presented in Table 1. 

**Table 1:** Atypical presentations of Hansen's disease ENL: Erythema nodosum leprosum; MBMDT: Multibacillary multidrug therapy; PBMDT: Paucibacillary multidrug therapy; AFB: Acid-fast bacilli.

Case	Age	Gender	Atypical presentation	Systemic Features	Referral department	Relation to MBMDT	Histopathology	Leprosy spectrum	Treatment	AFB
1	47	Male	Erythematous nodules, urticarial plaques with pseudovesicles on the upper extremities (mimicking Sweet’s syndrome)	Fever, malaise, body pain	General surgery	Not started	Granulomatous infiltration with foamy macrophages, lymphocytes, and neutrophils (suggestive of ENL)	Lepromatous leprosy with ENL	MBMDT with systemic steroids	3+
2	50	Male	Multiple erythematous nodules, nodulo-ulcerative lesions on the back	Fever, joint pains	General surgery	Not started	Granulomatous infiltration	Lepromatous leprosy with ENL	MBMDT with systemic steroids	3+
3	68	Male	Multiple ill-to-well-defined ulcers with jagged edges, necrotic slough, foul-smelling discharge, madarosis	-	Walk-in	Not started	Orthokeratosis in epidermis, acanthosis, dermal infiltration with foamy macrophages	Lucio phenomenon (lepromatous)	MBMDT with steroids	6+
4	39	Female	Erythematous, edematous plaques on photo-exposed areas (forehead, earlobes, forearms)	Fever	Walk-in	Not started	Granulomatous inflammation around neurovascular bundles with foamy macrophages, lymphocytes	Lepromatous leprosy with ENL	MBMDT with systemic steroids	3+
5	34	Male	Swelling and erythema on the feet, infiltrated plaque on the forehead	Fever, severe joint pains	General medicine	Discontinued after one month	Lymphohistiocytic infiltration around vessels, follicles, eccrine structures	Borderline lepromatous leprosy	MBMDT	No
6	48	Male	Ill-defined erythematous plaques on the cheek	-	Walk-in	Not started	Tuberculoid granulomatous lichenoid inflammation with lymphocytes, epitheloid cells, Langerhans cells	Tuberculoid leprosy	MBMDT	No
7	9	Male	Single hypopigmented patch on the cheek	-	-	Not started	Tuberculoid granuloma with lymphocytes, epitheloid cells in nerve twigs, superficial and deep lymphocytic infiltration	Tuberculoid leprosy	PBMDT	No
8	50	Male	Spontaneous ulceration on hands	-	-	Not started	Granulomatous inflammation	Lepromatous leprosy	MBMDT	3+
9	76	Male	Erythematous, annular plaques on thighs and hands	Fever	Pulmonology	Not started	Tuberculoid granulomatous inflammation around neurovascular structures	Tuberculoid leprosy	MBMDT with systemic steroids	No
10	23	Male	Grouped erythematous papules and nodules on lower back	Fever, joint pains	-	Not started	Lepromatous granuloma with foamy macrophages, neutrophils	Lepromatous leprosy with ENL	MBMDT with oral steroids	1+
11	30	Male	Multiple erythematous nodules, plaques with pseudovesicles on upper extremities	Fever, joint pains	-	Not started	Lepromatous granuloma with foamy macrophages, neutrophils	Lepromatous leprosy with ENL	MBMDT with oral steroids	4+
12	35	Male	Multiple asymptomatic lesions over body, including palms and soles	-	-	Not started	Histoid leprosy	Histoid leprosy	MBMDT	No
13	48	Male	Multiple asymptomatic nodular lesions on abdomen, back	Clawing of right hand	-	Not started	Histoid leprosy	Histoid leprosy	MBMDT	No
14	48	Male	Tingling, numbness in right lower leg	-	Neurology	Not started	Ultrasonography showed granuloma	Lepromatous leprosy	MBMDT	Neuritic
15	38	Male	Facial nerve palsy, bilateral foot drop	-	Neurology	Not started	Ultrasonography	Lepromatous leprosy	MBMDT	Neuritic

The classification of Hansen’s disease in this study varied from tuberculoid to polar lepromatous types, with only one patient having received prior antimycobacterial antibiotics for leprosy. Acid-fast bacilli (AFB) were observed in 12 out of the 15 cases on biopsy, with bacillary indices (BI) reported by the pathology lab ranging from 3+ to 6+. The patient with Lucio phenomenon demonstrated a BI of 6+, indicating a high bacillary load. These findings underscore the importance of microbiological evaluation in confirming the diagnosis and understanding the disease's severity.

**Figure 2:**
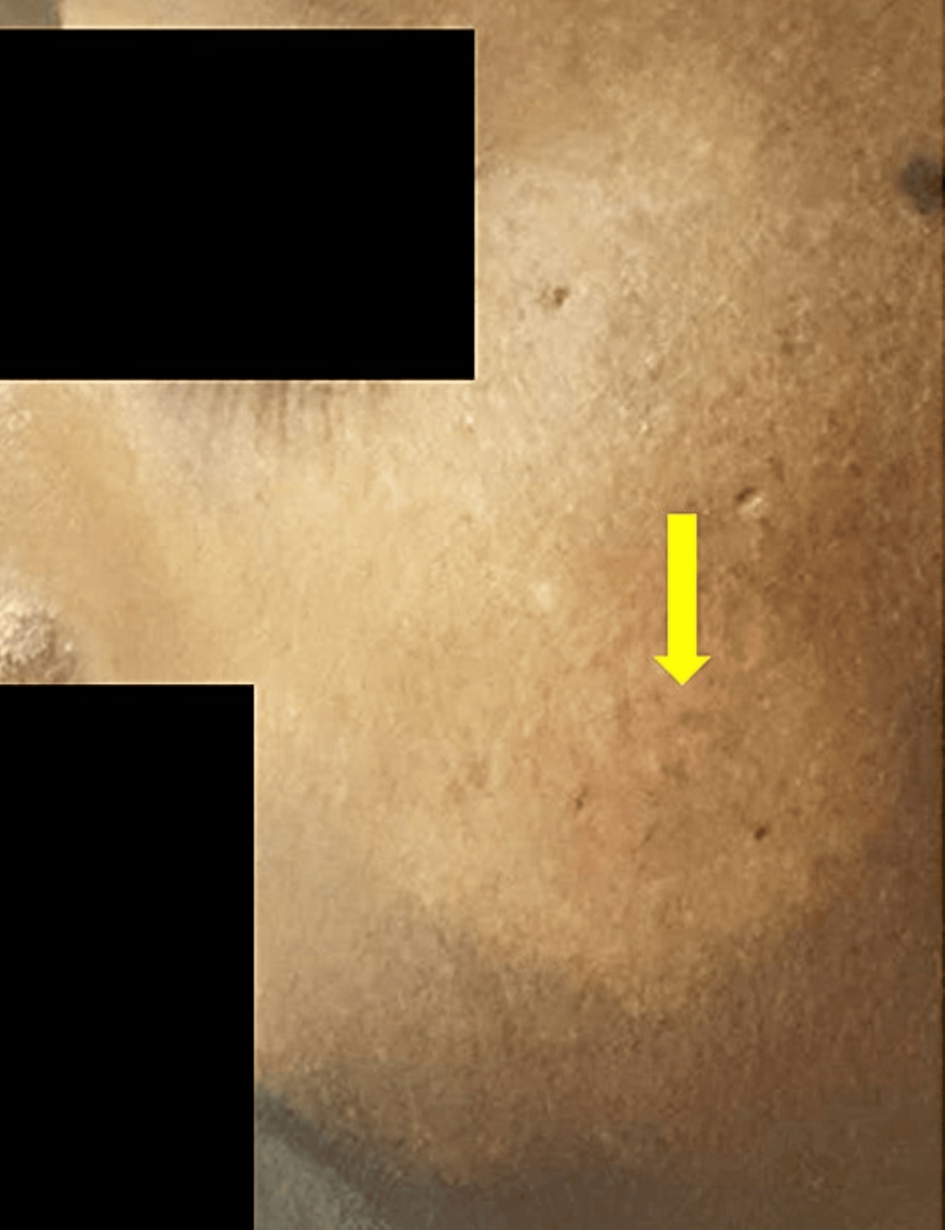
Indeterminate leprosy with a single, large, hypopigmented patch over the cheek

Three patients fell within the tuberculoid spectrum (cases 6, 7, and 9). One of them (case 9) presented with multiple itchy, annular lesions with central clearing and was diagnosed and treated as having granuloma annulare. The second patient (case 6) had a single, asymptomatic, hypopigmented macule on the right cheek, consistent with indeterminate leprosy. The third (case 7) exhibited a single large hypopigmented patch over the left cheek, which was initially misdiagnosed as pityriasis alba (Figure 2).

Another patient (case 1) experienced itchy, painful wheals on the cheek and bilateral extremities, closely mimicking urticarial vasculitis. Two patients (cases 14 and 15) were classified under the lepromatous spectrum, both referred from the neurology department. One of them (case 14) had swelling and erythema over both feet resembling erythromelalgia and infiltrated plaque over the forehead. The other (case 15) presented with spontaneous ulceration on both hands with no prior history of trauma and no lesions elsewhere on the body and was treated for non-healing ulcers.

Two patients presented with isolated nerve involvement without visible skin lesions. One patient had tingling and numbness in bilateral legs with sudden-onset partial clawing of the right hand. There was no prior trauma and no cutaneous lesions elsewhere. The second patient also had intermittent pain in the lower limbs and underwent a Doppler study and was treated for deep vein thrombosis. Ultrasound imaging in the pure neuritic Hansen’s disease cases revealed nerve edema consistent with neuritis, while an MRI demonstrated nerve thickening and inflammation without evidence of abscess formation or other structural abnormalities. These findings provided critical support for the diagnosis, highlighting the utility of advanced imaging techniques in confirming nerve involvement in atypical presentations of Hansen’s disease. Cases 14 and 15 presented as pure neuritic Hansen’s disease.

**Figure 3:**
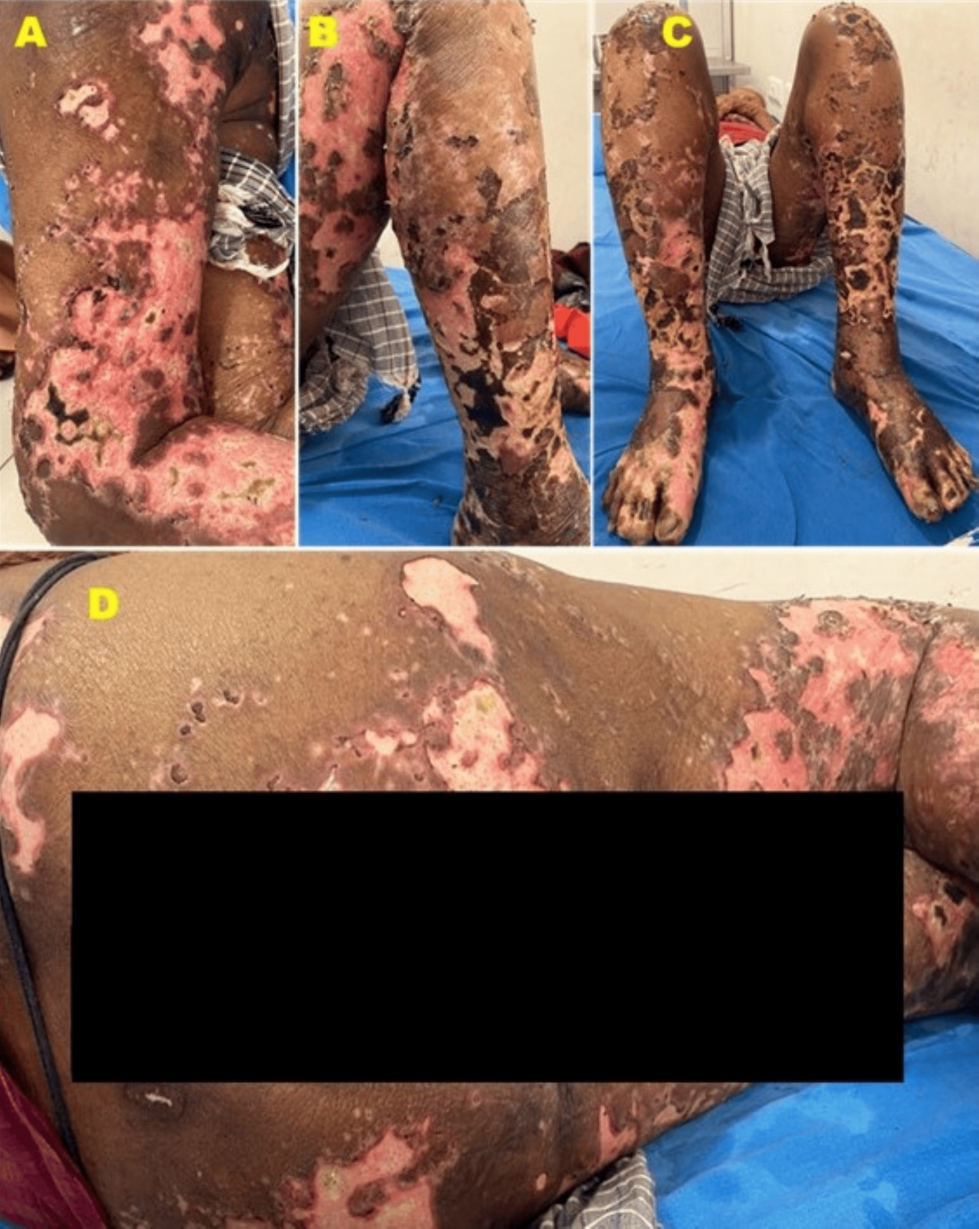
Many ulcers, some sloughing, seen on the arm (A), legs (B, C) and buttocks (D) of an untreated patient with Hansen's disease

Six patients were diagnosed with the ENL variant of leprosy. Among them, two patients presented with multiple, erythematous, tender nodules, pseudovesicles, and urticarial plaques typically associated with acute febrile neutrophilic dermatosis. One patient presented with multiple tender, polycyclic ulcers over the trunk and extremities like erythema nodosum necroticans, while another patient had multiple painless large ulcers over the trunk, abdomen, buttocks, and extremities with edema of both legs and was suspected of having vasculitis. Later on, a biopsy proved to be a Lucio phenomenon (Figure 3). The diagnosis was supported by the clinical presentation, including necrotic skin lesions, and histopathological findings, which confirmed the presence of abundant AFB, characteristic of the Lucio phenomenon.

One patient (case 4) had a history of photosensitivity with erythematous, edematous, nodules and plaques on sun-exposed areas, and she was treated as having photodermatitis before leprosy was diagnosed. Another presented with localized, recurrent, asymptomatic skin lesions. All six ENL patients displayed systemic symptoms, including fever and joint pains, which delayed the diagnosis of lepromatous leprosy. Two patients were diagnosed with histoid Hansen’s disease. One of them presented with multiple, asymptomatic, papulonodular, discrete lesions all over the body, including the torso and limbs, and was misdiagnosed as having molluscum contagiosum. The other had asymptomatic papules over the abdominal flank areas for a long duration and was diagnosed as having xanthoma. 

Out of the 15 patients, only one (case 5) received multibacillary multidrug therapy (MBMDT), and the antibiotics were discontinued after one month. The remaining patients had been initially misdiagnosed due to their atypical presentations and had not received MBMDT prior to their inclusion in this study. Following accurate diagnosis based on clinical evaluation, slit skin smear positivity, and histopathological confirmation, all patients were treated with MDT. For multibacillary (MB) cases, the regimen consisted of dapsone, rifampin, and clofazimine for a duration of 12 months, while paucibacillary (PB) cases received dapsone and rifampin for six months. Patients presenting with ENL (cases 4, 10, and 11) were additionally treated with systemic steroids, such as prednisolone, to manage severe inflammatory reactions, with the duration tailored to their clinical response. In some cases, adjunctive antibiotics were administered to address secondary infections, and physiotherapy was provided for nerve-related complications, particularly in pure neuritic cases. This comprehensive approach ensured that the treatment was individualized based on the clinical presentation and severity of each case. In a few cases, systemic antibiotics were also given for a brief period. In the pure neuritic Hansen’s cases, the patients underwent physiotherapy along with MBMDT.

## Discussion

Leprosy is an infectious disease where clinical symptoms are significantly influenced by the patient’s immune response, making it a valuable model for studying the host's defense against intracellular pathogens [4-8]. The variability in immune response contributes to diverse presentations of acute inflammatory episodes known as "leprosy reactions," which can cause nerve damage requiring urgent medical attention [12]. In this study, all the cases of Hansen’s disease presented initially with type-2 inflammatory reactions, a common feature of multibacillary forms. Atypical skin manifestations included infiltrated plaques on cooler, air-exposed areas, spontaneous ulcerations of histoid Hansen’s disease, erythema multiforme-like lesions, and Blaschkoid patterns.

MBMDT remains the cornerstone of treatment but could be optimized for atypical presentations by adjusting drug combinations and duration based on disease severity and the bacillary load [12]. Adjunct therapies, such as clofazimine and thalidomide, are not only effective against Mycobacterium leprae and Mycobacterium lepromatosis but also act as potent anti-inflammatory agents in the management of ENL. Systemic steroids, while effective in controlling severe inflammatory responses like ENL, carry risks, highlighting the need for careful monitoring and gradual tapering to minimize adverse effects [12].

In pure neuritic Hansen’s disease, high-resolution ultrasonography (HRUS) proved valuable for identifying nerve thickening and inflammation when traditional methods were inconclusive. Standardizing HRUS as a diagnostic tool could improve early detection and treatment outcomes. Misdiagnoses such as Sweet’s syndrome, urticarial vasculitis, and deep vein thrombosis were delayed in 14 of these 15 exacerbating symptoms and increased complications like nerve damage and deformities. 

Public health strategies in hyperendemic regions should include strengthening early detection programs, increasing awareness among healthcare workers about atypical presentations, and promoting timely initiation of MDT. Community-based screening, enhanced contact tracing, and education campaigns are essential. Investigating genetic predispositions, such as polymorphisms in immune regulatory genes [e.g., tumor necrosis factor-alpha (TNF-α), human leukocyte antigen (HLA), toll-like receptor 2 (TLR2)], may help identify at-risk populations and improve diagnostic and therapeutic strategies [12].

Histoid leprosy, described by Wade in 1960, presents with distinct nodular lesions and spindle-shaped cells in untreated cases [13]. While untreated patients exhibit diffuse papulonodular lesions, those with a history of anti-leprosy treatment may develop more localized lesions due to bacterial resistance or immune modulation. The high bacillary load in atypical cases, including histoid and ENL variants, underscores the importance of early diagnosis and treatment to prevent community transmission [14-16].

Six of 15 patients were referred from other departments due to systemic manifestations and deformities. Clinicians need to recognize how leprosy mimics other dermatological and neurological conditions, delaying the treatment of a treatable disease. For example, two cases of pure neuritic Hansen’s disease presented with chronic nerve pain, tingling, and deformities. Leprosy-related neuritis is a medical emergency due to its potential to cause rapid nerve damage, sensory and motor disabilities, and deformities if untreated [17,18]. Physiotherapy plays a vital role in preventing deformities by maintaining muscle strength and joint mobility and preventing contractures. Early physiotherapy, combined with MDT, slows nerve damage progression and aids recovery [17,18]. These findings underscore the need for higher clinical vigilance in endemic areas and multidisciplinary collaboration for early detection.

Limitations

This study has limitations. First, the sample size was relatively small, limiting the generalizability of the findings. Second, the study was conducted in a single tertiary care centre, which may not represent the full range of presentations in other regions. Additionally, the data collection was retrospective, relying on patient records, which may not capture all aspects of clinical presentation and progression. Finally, the lack of follow-up data limits our understanding of the long-term outcomes for these atypical cases.

## Conclusions

Early detection and timely treatment are essential for controlling leprosy and achieving its eradication. The disease’s ability to mimic dermatological and neurological conditions presents significant diagnostic challenges, requiring healthcare professionals, especially in endemic regions, to appreciate its diverse presentations. All vitiligo-like skin lesions and plaques in endemic areas should be screened for light touch sensation using a cotton swab. If anesthesia is detected, a biopsy should be performed to check for neuritis and AFB, and treatment should be initiated if positive. Regular training, standardized referral protocols, and integrating leprosy awareness into medical education are critical for improving early recognition of atypical cases. Our findings, consistent with regional and global studies, highlight the prevalence of misdiagnoses, delayed treatment, and atypical manifestations such as Sweet’s syndrome-like lesions and the Lucio phenomenon. Regional differences in healthcare access, awareness, and genetic factors further influence these manifestations, underscoring the need for comparative studies to inform public health strategies. Raising awareness and promoting early medical attention among at-risk populations are vital to preserving peripheral nerves and preventing deformities and disabilities among patients with leprosy.
